# Vortex vein cauterization and truncation to avoid perfluorocarbon syndrome during endoresection of uveal melanomas: a retrospective study

**DOI:** 10.1038/s41433-022-02119-x

**Published:** 2022-05-30

**Authors:** Miltiadis Fiorentzis, Nikolaos E. Bechrakis

**Affiliations:** University Hospital Essen, Department of Ophthalmology, University of Duisburg-Essen, Hufeland Str. 55, 45147 Essen, Germany

**Keywords:** Surgery, Adverse effects

## To the Editor:

We read with great interest the brief communication “Perfluorocarbon syndrome–a possible, overlooked source of fatal gas embolism following uveal melanoma endoresection” by Ruschen et al. [1]. The authors hypothesize gas embolism due to the entry of perfluorooctane (PFO) in the bloodstream, the formation of gas bubbles in the pulmonary circulation, and the association with a higher vapor pressure of PFO. We present a modification of the operating technique during endoresection of large uveal melanomas, which minimizes the intraoperative egress of perfluorocarbon liquid (PFCL) into the circulatory system.

**Fig. 1:**
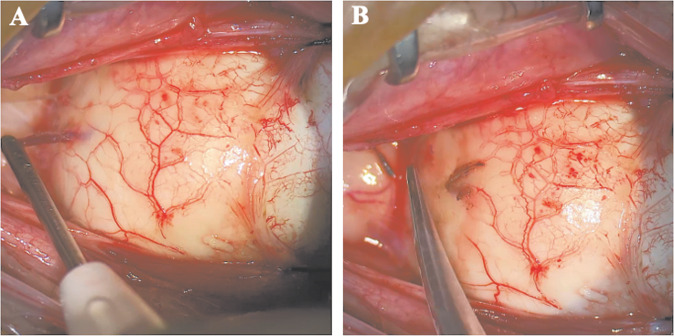
Vortex vein cauterization and truncation at the tumor base. **A** Localization and cauterization of the vortex vein at the tumor base. **B** Truncation of the votex vein following cauterization.

From January 2021 until April 2022, 53 patients with large uveal melanoma undergoing an adjuvant endoresection after either gamma knife or stereotactic irradiation were analyzed. Endoresection was conducted without truncation of the vortex veins in 29 patients (54.7%) until August 2021. In this group, PFCL drained away in ten patients (34.4%) and additional one to three bottles had to be administered intraoperatively due to an apparent lower level of PFCL during laser retinopexy and before direct PFCL-silicon oil exchange. In 24 patients (45.3%) operated after August 2021, the vortex veins were cauterized and truncated in the corresponding quadrant(s) of the tumor base at the beginning of the procedure (Fig. 1A, B). A leakage of PFCL was not observed in any of these cases.

We hypothesize that PFCL egressed out of the eye in the first group of this series through the sclera via the tearing of the vortex veins and its ampullae due to high intraocular pressure (IOP). During endoresection, the tumor excision must be conducted under elevated IOP up to 80 mmHg, which leads to a venous compression including the vortex veins. When the choroid is excised up to the sclera, the integrity of the veins may be preserved through the intact surrounding sclera, allowing PFO to escape [2]. We have never used fluid-air exchange during endoresection to avoid air venous embolization but have commonly observed the lowering of the PFCL level in the eye during surgery, presumably due to egress of the fluid via the vortex veins [3]. The egress of the fluid is foremost enabled through an elevated IOP and choroidal veins cut flush to the ampullae in the sclera. We always use perfluordecalin and have never experienced any serious adverse event, even in the patients with significant PFCL egress during surgery [4].

The vortex veins can be large with thin walls expanding under higher pressure, therefore rapidly enabling air entrainment or liquid egress and fatal vascular air embolism [4]. The truncation of the corresponding vortex veins just before endoresection may constitute a further precaution to avoid or significantly reduce the risk of gas embolism due to PFCL entry in the venous circulation.
